# Adipocytes, Innate Immunity and Obesity: A Mini-Review

**DOI:** 10.3389/fimmu.2021.650768

**Published:** 2021-06-24

**Authors:** Alecia M. Blaszczak, Anahita Jalilvand, Willa A. Hsueh

**Affiliations:** Hsueh Laboratory, The Ohio State University Wexner Medical Center, Diabetes and Metabolism Research Center, Columbus, OH, United States

**Keywords:** adipocytes, obesity, adipose tissue, inflammation, innate immunity

## Abstract

The role of adipose tissue (AT) inflammation in obesity and its multiple related-complications is a rapidly expanding area of scientific interest. Within the last 30 years, the role of the adipocyte as an endocrine and immunologic cell has been progressively established. Like the macrophage, the adipocyte is capable of linking the innate and adaptive immune system through the secretion of adipokines and cytokines; exosome release of lipids, hormones, and microRNAs; and contact interaction with other immune cells. Key innate immune cells in AT include adipocytes, macrophages, neutrophils, and innate lymphoid cells type 2 (ILC2s). The role of the innate immune system in promoting adipose tissue inflammation in obesity will be highlighted in this review. T cells and B cells also play important roles in contributing to AT inflammation and are discussed in this series in the chapter on adaptive immunity.

## Introduction

Obesity is a growing healthcare problem in the United States and globally. It is a leading cause of preventable death and currently impacts more than 35% of the US population (1). This number is estimated to rise with a projected 42% of the US adult population being obese by 2030 (2). Obesity adversely impacts the entire body leading to increased Type 2 diabetes and associated complications, Alzheimer’s disease, vascular dementia, obstructive sleep-apnea, accelerated atherosclerosis, heart failure, fatty liver disease, nonalcoholic steatohepatitis, osteoarthritis, altered immune system, impaired response to vaccines, and increased susceptibility to cancer compared to aged-matched lean individuals (3). Obesity is characterized by an expansion of both visceral and subcutaneous adipose tissue (AT) in the setting of chronic over-nutrition. The ensuing chronic low-grade inflammation sets the stage for many of the extensive complications. Thus, understanding mechanisms that mediate the immunological changes in obesity may unlock new therapeutic strategies. This review places a special emphasis on the innate immune system and the adipocyte.

## Innate and Adaptive Immunity

The role of the immune system is to identify self- versus non-self to eliminate potential toxins, allergens, and pathogens without destroying the host tissue. The immune system is composed of two key functional responses, innate and adaptive immunity. The innate immune response is the initial line of defense after the host’s external barrier. This immune response is not antigen-specific, but rather recognizes molecular patterns that are inherent to the toxin, allergen, or pathogen. This allows for the rapid activation of an immune response which is followed by the development of an antigen-specific immune response. The adaptive immune system upon the first encounter of a toxin, allergen, or pathogen undergoes expansion to aid the innate immune response. Upon resolution, a subset of these adaptive immune cells persists and creates a distinct population of memory immune cells. Memory cells are faster to respond to future encounters with the same pathogen, allergen, or toxin. The cellular component of both immune responses arises from the hematopoietic stem cell. This pluripotent cell further differentiates within the bone marrow to generate either the common lymphoid progenitor or the common myeloid progenitor. The common myeloid progenitor gives rise to lineage-specific colony-forming cells which then further develop into a majority of the innate immune cells as well as megakaryocytes (platelets) and erythrocytes (red blood cells). The cellular components of the innate immune response include the granulocytes including monocytes, macrophages, neutrophils, basophils, eosinophils, mast cells, and dendritic cells, as well as adipocytes. While the adipocyte is not traditionally viewed as an immune cell, recent research has demonstrated that the adipocyte releases adipokines, microRNAs and lipids to influence the innate immune response (4–7). The adipocyte also expresses MHCII molecules during high-fat diet feeding allowing the adipocyte to interact with naïve T cells resulting in T cell differentiation and activation (8, 9). The common lymphoid progenitor gives rise to the key immune cells within the adaptive immune response including B cells and T cells as well, as more recently discovered innate immune cells including the NK cell and the innate lymphoid cell types 1, 2, and 3 (10, 11). The key link between the innate and adaptive immune system is antigen presentation.

## Inflammation in Adipose Tissue; Contribution of Innate Immunity

The AT immune cell microenvironment in the lean state is a well-balanced crosstalk between the adipocyte and the stromal vascular fraction (SVF) or the cellular compartment of AT. In lean mice, the SVF is comprised of mesenchymal stem cells (12), endothelial progenitor cells (13–16) as well as numerous immune cells including anti-inflammatory immunoregulatory T cells, Tregs (17), innate lymphoid type 2 cells (ILC2) (18), alternatively activated macrophages (19, 20), and eosinophils (21). These cells work in concert to ensure the maintenance of homeostasis within AT including maintaining systemic insulin sensitivity. However, upon high-fat diet (HFD) feeding, there is a disruption of the anti-inflammatory milieu with increased differentiation and recruitment of pro-inflammatory immune cells creating a chronic, low-grade inflammatory state. In murine obesity, AT is characterized by the early, transient infiltration of neutrophils (22) followed by the accumulation of pro-inflammatory CD8+ T cells (23), CD4 Th1 cells (8) and M1 macrophages (19, 20) all of which surround the dying adipocyte forming a crown-like structure. Innate immunity is an early and key component in sustaining AT inflammation.

### Adipocyte

The adipocyte, unlike most traditional immune cells, links the innate and adaptive immune systems through adipokine, lipid and exosome release and through antigen presentation. While the adipocyte is the primary site of energy storage for the body and performs multiple metabolic activities, it can assume the role of a highly functional immune cell, releasing anti- and pro-inflammatory cytokines and hormones (adipokines), as well as lipids, which also act as signaling molecules (24). Since its discovery as an endocrine cell, the adipocyte has been identified to secrete more than 50 adipokines/cytokines including adiponectin (4, 25), leptin (5), TNFα (26–29), visfatin (30), and resistin (31) among many others (32) which impact local and systemic metabolism and inflammation.

The first adipokine described was leptin which revolutionized our understanding of the critical role that adipocytes play in whole-body energy homeostasis (33). Mutations in the leptin gene in the ob/ob mouse model led not only to hyperphagia and weight gain but also disruptions in fertility and body temperature regulation (34). Treatment of ob/ob mice with recombinant leptin, but not db/db (leptin receptor-deficient) mice, led to improved body weight and decreased food intake (35). However, contrary to what was initially hypothesized, leptin is found in higher levels in obese patients as compared to lean controls (36) suggesting the presence of leptin resistance (37). Increasing evidence supports an immunologic role of leptin. Leptin deficiency is associated with greater susceptibility to death after administration of LPS or TNFα which is partially corrected with leptin administration (38, 39). Macrophages from leptin-deficient mice have impaired phagocytosis and altered cytokine production (40, 41). In neutrophils, leptin appears to increase ROS production (42), inhibit apoptosis (43), and affect neutrophil migration (44) suggesting that leptin impacts cells that mediate the innate immune response. More recently, Scherer and colleagues (45) demonstrated that hyperleptinemia is a driving force for metabolic disorders. Interestingly, a partial decrease of circulating leptin in obesity reestablishes hypothalamic leptin sensitivity and effectively reduces weight gain and enhances insulin sensitivity.

Unlike leptin, adiponectin, a key adipokine involved in energy homeostasis, is reduced in obese subjects and has anti-inflammatory effects. Adiponectin is found in higher levels in AT and blood of lean subjects (46). Ob/ob mice with adiponectin overexpression have an increased ability to expand their subcutaneous AT associated with in a reduction of systemic and local AT inflammation. These mice also develop less ectopic lipid deposition in the liver and skeletal muscle leading to improvements in insulin sensitivity despite greater amounts of AT (47). Within the innate immune system, adiponectin acts primarily on macrophages resulting in a greater polarization of M2-like macrophages, decreased M1-like macrophages, and a reduction in ROS production (48). In neutrophils, adiponectin functions to decrease the production of the neutrophil chemokine CXCL8 (49) and ROS *via* modulation of NADPH oxidase (50). These observations highlight the yin-yang relationship of leptin and adiponectin, which functions as an anti-inflammatory regulator of the innate immune response. These hormones are the most well-known adipokines, but as discussed above, numerous other have been identified. Consistently obese versus lean humans and mice reveal increased proinflammatory and extracellular matrix gene expression, but the function of many adipokines remains unknown (51, 52).

Lipid release is another important mechanism by which adipocytes can impact immune cells in the AT microenvironment. Lipids such palmitate and other unsaturated fatty acids can bind to toll-like receptors (TLRs) on the surface of immune cells, such as macrophages, and are converted to ceramides and diacylglycerols during states of lipid overabundance as occurs in obesity. These toxic lipids enhance proinflammatory signaling (7). More recently, branched-chain fatty acid esters of hydroxyl fatty acids (FAHFAs) produced by adipocytes were shown to bind to G-protein coupled receptors (GPRs) 40 and 120 to inhibit inflammation and improve insulin secretion and sensitivity (53). Within the blood and subcutaneous adipose tissue of insulin-resistant humans and mice, there is a reduction of several FAHFAs most notably palmitic acid esters of hydroxyl stearic acids. Supplementation of these *via* oral ingestion or subcutaneous administration improves glucose and insulin handling (53). These observations indicate adipocytes release both pro- and anti-inflammatory lipids as a mechanism to modulate the immune system.

Adipocytes are composed of large unilocular lipid droplets containing triacylglycerols and neutral free fatty acids (FFAs), which are a major mechanism for energy storage and release. Adipocytes not only release lipids and secrete adipokines, but they can also employ exosomes- extracellular vesicles (40-150 nm in size) of endosomal origin to participate in this process. Exosomes are increasingly recognized as a novel mechanism by which adipocytes communicate with other cells and target tissues. Their cargo contains adipokines, lipids, and microRNAs. Release is dependent on nutritional status and degrees of adiposity: increased release in obesity and decreased release with caloric restriction or lipodystrophy. Exosomes can be taken up by endocytosis, pinocytosis, or phagocytosis, and can be directed to target cells by adhesion molecules on the exosomal surface (6). Recently, Flaherty, et al. reported that adipocytes of mice release 1% of their lipid content daily ex vivo *via* exosomes, which was increased in obese mice. This release of exosomes contributed to macrophage foam cell formation, suggesting that exosomes contribute to the orchestration of AT immune cells (54). MicroRNAs, which regulate protein translation, are another important component of adipocyte-derived exosomes. Adipocytes are a major source of microRNAs in the circulation with greater than 55 differentially expressed microRNAs between lean and obese individuals. Adipocyte microRNAs contribute to the regulation of metabolism, inflammation, and multiple biologic processes locally and systemically (55). However, controversy exists as to whether adipocyte exosomes represent a minority or majority of circulating exosomes (54, 56).

Finally, one of the most unique features of the adipocyte is its ability to function as an antigen-presenting cell, which is described in detail in Chapter XX of this series by Deng et al. The adipocyte can present antigen to promote differentiation and activation of interferon gamma-producing CD4 Th1 cells. This activity is increased early in obesity, after only 2 weeks HFD in mice, before the AT macrophage increase, suggesting adipocytes both instigate and maintain AT inflammation (8). Adipocytes provide a critical link between the innate and adaptive immune systems.

### Neutrophils

Neutrophils are one of the initial inflammatory cells recruited to sites of host injury. As a component of the innate immune system, neutrophils have four primary activities including, phagocytosis, degranulation, reactive oxygen species (ROS) production, and neutrophil extracellular trap (NET) formation (57). Within mouse peripheral blood, neutrophils comprise 10-25% of the circulating immune cells (58), whereas in humans they compromise 50-70% of circulating immune cells. Furthermore, unlike humans, mouse neutrophils do not have defensins (anti-microbial peptides). While there are several differences between mouse and human neutrophils, mouse models are still routinely used for genetic manipulation (59).

Early studies in mice observed the transient infiltration of neutrophils into the AT after the start of HFD resulting in maximal levels by day 3 and undetectable levels by day 28 (22). However, others have suggested a more prolonged presence, contributing to about 2% of the SVF (60). Despite the early and relatively small contribution of the neutrophil in obese mice, loss of neutrophil elastase (60) or myeloperoxidase (61) leads to a decrease in AT inflammation and macrophage recruitment and promotes resolution of insulin resistance. However, loss of neutrophil NET formation does not impact obesity-related inflammation or insulin resistance in HFD-fed mice (62).

Within human AT, there have been fewer studies. One identified the presence of neutrophils within subcutaneous AT and reported that neutrophils were contained within the vasculature with no or limited infiltration into the tissue similar to vascular pools of neutrophils found in the liver (63). Another study showed limited infiltration in obesity in both visceral and subcutaneous AT (64); however, the quantity, cause of recruitment, and function of these cells within human AT remains unknown. Despite this, inflammatory lipids such as leukotrienes are known to attract and activate neutrophils. Under inflammatory conditions adipocyte and macrophages produce increased IL-8, a powerful neutrophil chemoattractant (65, 66). Additionally neutrophils can self-recruit *via* increased production of CXCL2, another known neutrophil chemoattractant (67). Using a mouse peritonitis model, Tynan et al. demonstrated that lipids extracted from human adipocytes promoted migration and accumulation of neutrophils and macrophages, and activated these cells to produce cytokines (68). These effects were similar whether the adipocytes were obtained from lean or obese subjects, as fatty acid profiles, analyzed by gas chromatography, were not different. Oleic acid was also shown to recruit neutrophils in a similar mouse model (69). Additionally, adipocyte lipolysis has been shown to attract neutrophils and enhance their production of IL-1β leading to the activation of adipocytes and other immune cells (70). Further studies will be useful to determine the types of adipocyte lipids and other factors that attract and activate neutrophils into AT.

### Macrophages

One of the most well-studied immune cells in AT and a key component of the innate immune response is the macrophage. Within mouse models of obesity, macrophages comprise up to 40% of the SVF (71) and are shown to be involved in the development of insulin resistance (72), atherosclerosis progression (73), and other obesity-related complications. In mice, macrophages have been classified into the relatively simplistic M1 and M2 phenotypes with obesity increasing the prevalence of pro-inflammatory M1-like macrophages (19). Ablation of these CD11c+ proinflammatory macrophages decreases AT inflammation and interferes with the development of insulin resistance, suggesting that macrophages are key mediators of insulin sensitivity (72). Furthermore inhibiting macrophage recruitment through the genetic depletion of CCR2 (74) or MCP-1 (75) also leads to the repression of AT inflammation and insulin resistance during HFD feeding. In contrast, accumulation of anti-inflammatory PPARγ positive macrophages (M2 macrophages) leads to improvements in AT inflammation and insulin sensitivity (76), while loss of macrophage PPARγ increases AT inflammation and insulin resistance (77). The balance between the pro and anti-inflammatory macrophage subtypes is much less defined within human AT with most AT macrophages expressing both common M1 and M2 markers (78, 79). Although there is still some debate on this topic with others suggesting a greater abundance of CD206 macrophages after weight loss (80). Further research on human adipose tissue macrophage subsets still needs to be done. The interaction between adipocytes and ATMs begins with the formation of crown-like structures characterized by macrophage accumulation surrounding dying adipocytes. This process is mediated by the adipocyte secretion of MCP-1 resulting in macrophage accumulation and activation (81). Once accumulated, these macrophages release TNFα which increases adipocyte release of FFAs (82). FFAs are capable of binding TLR4 on both the adipocyte and the macrophage resulting in NFkB activation and release of IL1β by macrophages (83). Adipocyte turnover occurs with approximately 10% of adipocytes undergoing apoptosis annually (84). These dying adipocytes are removed *via* trogocytosis by ATMs (85). Adipokine secretion by adipocytes also alters macrophage function. Increased leptin in obesity increases the phagocytic function of ATMs and is associated with an increase in circulating C-reactive protein (41, 86). Adiponectin secretion is thought to be inhibited by TNF-alpha secretion which is increased in obesity. In the lean state, adiponectin is known to inhibit the development of foam cells from macrophages and decreases endothelial cell activation and monocyte adhesion (87). The interaction between adipocytes and ATMs is a key contributor to the chronic low-grade inflammation of obesity (88).

### Innate Lymphoid Cells Type 2

Another key innate immune cell that is not well-defined within AT is the innate lymphoid cell type 2 (ILC2). ILC2 cells are important in the maintenance of insulin sensitivity and are decreased in the setting of HFD. Innate lymphoid cells express CD4+ related cytokines, mirror Th1, Th2, and Th3 expression profiles (89), but differ in that they do not express B or T cell receptors despite arising from the common lymphoid progenitor cell (90). The ILC2 cell is similar to Th2 cells in that it contains the transcription factor GATA3 (91) and secretes IL5 and IL13. Within mouse models of obesity, the administration of IL25 leads to improvements in glucose tolerance and weight loss and is associated with the infiltration of ILC2 cells, alternatively activated macrophages, and eosinophils. Depletion of ILC2 cells in obese Rag1-/- mice leads to worsening insulin sensitivity and weight gain, while repletion of ILC2 cells reverses these negative metabolic consequences (18). Furthermore, in murine models, ILC2 cells appear to be the primary source of IL-5 and IL-13 and are necessary for the maintenance of alternatively activated macrophages and eosinophils, two key cells implicated in the anti-inflammatory state of lean AT (92). These cells are thought to contribute to an improved metabolic phenotype through the beiging of WAT characterized by increased expression of Ucp1 leading to an increase in caloric expenditure and attenuation of weight (93, 94). These researchers also confirmed that ILC2 cells are markedly decreased in the SAT of obese compared to lean humans (93); thus highlighting a key role for ILC2 cells in AT (95).

## Conclusions

**Figure 1:**
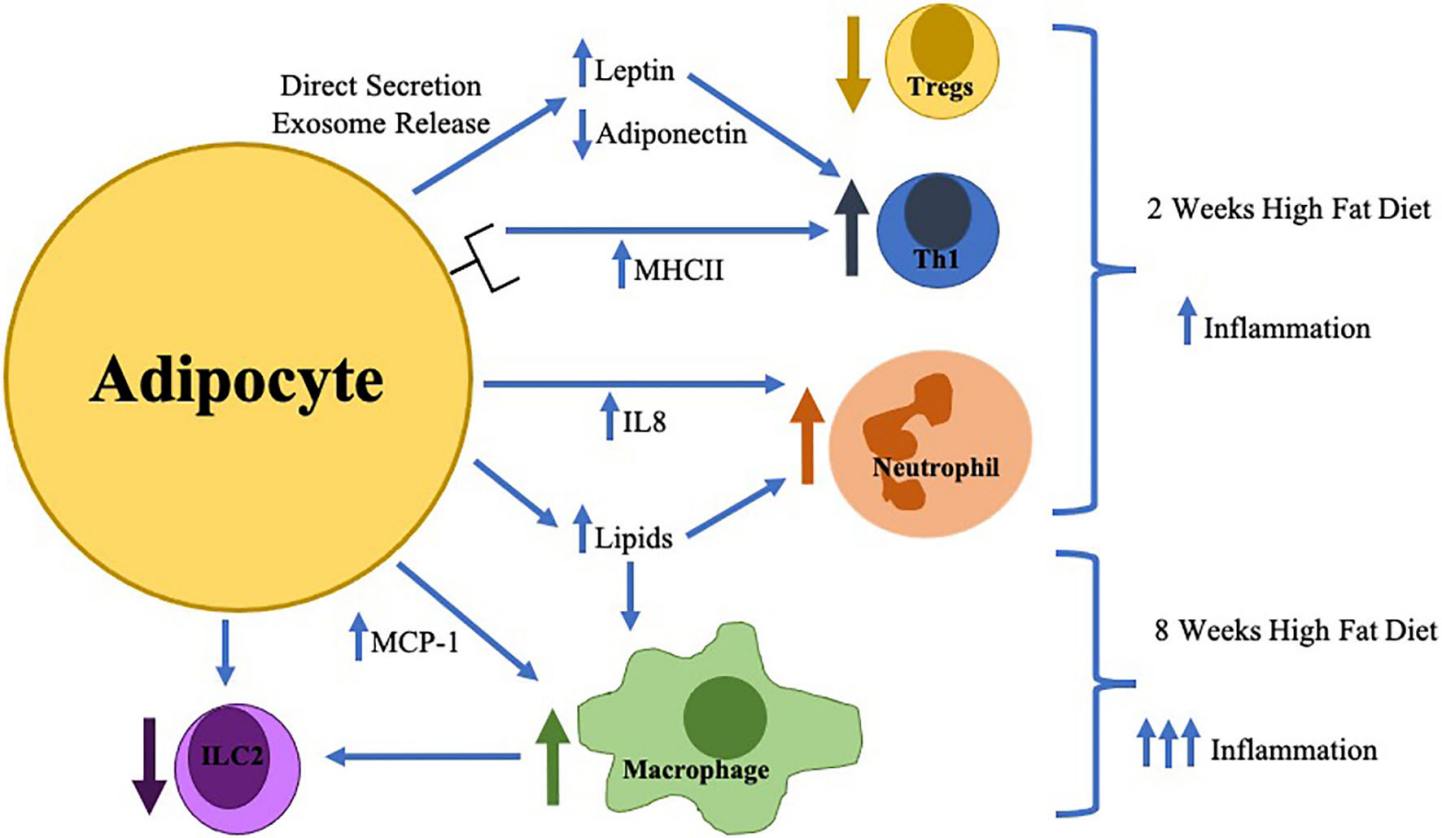
The earliest changes in adipose tissue inflammation include adipocyte hypertrophy with direct secretion/exosome release of key adipokines (increased leptin/decreased adiponectin) which in combination with increased adipocyte MHCII expression result in the differentiation of pro-inflammatory Thelper type 1 cells (Th1) resulting in decreased regulatory T cells (Tregs). Adipocytes during this time frame also increase IL-8 production resulting in neutrophil accumulation. Later in the time course (around 8 weeks), macrophage infiltration is stimulated by toxic lipid production and increased MCP-1 secretion which inhibits innate lymphoid cell type 2 (ILC2) differentiation.

Multiple changes in the innate immune system are key contributors to inflammation in obese AT resulting in the development of obesity-related diseases as summarized in **Figure 1**. Despite the greater understanding of the immunologic role of AT, further investigation should seek to answer the following questions: (1) what are the causes of AT immune cell infiltration and activation, (2) how does the adipocyte contribute to these changes and interact with AT immune cells, (3) is there a role of the gut microbiota in alteration of AT inflammation and (4) how do AT immune cells change during weight-loss. Through the elucidation of the answers to these key questions, immunologic therapies, potentially targeting the adipocyte, for the treatment of obesity and its inflammatory complications can be developed.

## Author Contributions

AB, AJ, and WH were involved in all aspects of writing, editing, and decision to publish. All authors contributed to the article and approved the submitted version.

## Funding

Funding for this manuscript includes the American Diabetes Association (1-16-ICTS-049) and The National Institutes of Health (R01HL135622).

## Conflict of Interest

WH advises for Novo Nordisk and Intercept Pharmaceuticals.

The remaining authors declare that the research was conducted in the absence of any commercial or financial relationships that could be construed as a potential conflict of interest.

## References

[1] Flegal KM Kruszon-Moran D Carroll MD Fryar CD Ogden CL. Trends in Obesity Among Adults in the United States, 2005 to 2014. JAMA (2016) 315(21):2284–91. 10.1001/jama.2016.6458 PMC11197437 27272580

[2] Finkelstein EA Khavjou OA Thompson H Trogdon JG Pan L Sherry B. Obesity and Severe Obesity Forecasts Through 2030. Am J Prev Med (2012) 42(6):563–70. 10.1016/j.amepre.2011.10.026 22608371

[3] Kanneganti TD Dixit VD. Immunological Complications of Obesity. Nat Immunol (2012) 13(8):707–12. 10.1038/ni.2343 22814340

[4] Scherer PE Williams S Fogliano M Baldini G Lodish HF. A Novel Serum Protein Similar to C1q, Produced Exclusively in Adipocytes. J Biol Chem (1995) 270(45):26746–9. 10.1074/jbc.270.45.26746 7592907

[5] Maffei M Halaas J Ravussin E Pratley RE Lee GH Zhang Y. Leptin Levels in Human and Rodent: Measurement of Plasma Leptin and Ob RNA in Obese and Weight-Reduced Subjects. Nat Med (1995) 1(11):1155–61. 10.1038/nm1195-1155 7584987

[6] Akbar N Azzimato V Choudhury RP Aouadi M. Extracellular Vesicles in Metabolic Disease. Diabetologia (2019) 62(12):2179–87. 10.1007/s00125-019-05014-5 PMC6861353 31690986

[7] Schilling JD Machkovech HM He L Sidhu R Fujiwara H Weber K. Palmitate and Lipopolysaccharide Trigger Synergistic Ceramide Production in Primary Macrophages. J Biol Chem (2013) 288(5):2923–32. 10.1074/jbc.M112.419978 PMC3561515 23250746

[8] Deng T Lyon CJ Minze LJ Lin J Zou J Liu JZ. Class II Major Histocompatibility Complex Plays An Essential Role in Obesity-Induced Adipose Inflammation. Cell Metab (2013) 17(3):411–22. 10.1016/j.cmet.2013.02.009 PMC3619392 23473035

[9] Deng T Liu J Deng Y Minze L Xiao X Wright V. Adipocyte Adaptive Immunity Mediates Diet-Induced Adipose Inflammation and Insulin Resistance by Decreasing Adipose Treg Cells. Nat Commun (2017) 8(1):15725. 10.1038/ncomms15725

[10] Chaplin DD. Overview of the Immune Response. J Allergy Clin Immunol (2010) 125(2 Suppl 2):S3–23. 10.1016/j.jaci.2009.12.980 20176265 PMC2923430

[11] Walker JA Barlow JL McKenzie ANJ. Innate Lymphoid Cells — How Did We Miss Them? Nat Rev Immunol (2013) 13:75. 10.1038/nri3349 23292121

[12] Zuk PA Zhu M Ashjian P De Ugarte DA Huang JI Mizuno H. Human Adipose Tissue Is a Source of Multipotent Stem Cells. Mol Biol Cell (2002) 13(12):4279–95. 10.1091/mbc.e02-02-0105 PMC138633 12475952

[13] Miranville A Heeschen C Sengenes C Curat CA Busse R Bouloumie A. Improvement of Postnatal Neovascularization by Human Adipose Tissue-Derived Stem Cells. Circulation (2004) 110(3):349–55. 10.1161/01.CIR.0000135466.16823.D0 15238461

[14] Cai L Johnstone BH Cook TG Liang Z Traktuev D Cornetta K. Suppression of Hepatocyte Growth Factor Production Impairs the Ability of Adipose-Derived Stem Cells to Promote Ischemic Tissue Revascularization. Stem Cells (2007) 25(12):3234–43. 10.1634/stemcells.2007-0388 17901400

[15] Rehman J Traktuev D Li J Merfeld-Clauss S Temm-Grove CJ Bovenkerk JE. Secretion of Angiogenic and Antiapoptotic Factors by Human Adipose Stromal Cells. Circulation (2004) 109(10):1292–8. 10.1161/01.CIR.0000121425.42966.F1 14993122

[16] Sumi M Sata M Toya N Yanaga K Ohki T Nagai R. Transplantation of Adipose Stromal Cells, But Not Mature Adipocytes, Augments Ischemia-Induced Angiogenesis. Life Sci (2007) 80(6):559–65. 10.1016/j.lfs.2006.10.020 17157325

[17] Eller K Kirsch A Wolf AM Sopper S Tagwerker A Stanzl U. Potential Role of Regulatory T Cells in Reversing Obesity-Linked Insulin Resistance and Diabetic Nephropathy. Diabetes (2011) 60(11):2954–62. 10.2337/db11-0358 PMC3198056 21911743

[18] Hams E Locksley RM McKenzie AN Fallon PG. Cutting Edge: IL-25 Elicits Innate Lymphoid Type 2 and Type II NKT Cells That Regulate Obesity in Mice. J Immunol (2013) 191(11):5349–53. 10.4049/jimmunol.1301176 PMC3847854 24166975

[19] Lumeng CN Bodzin JL Saltiel AR. Obesity Induces a Phenotypic Switch in Adipose Tissue Macrophage Polarization. J Clin Invest (2007) 117(1):175–84. 10.1172/JCI29881 PMC1716210 17200717

[20] Lumeng CN DelProposto JB Westcott DJ Saltiel AR. Phenotypic Switching of Adipose Tissue Macrophages With Obesity Is Generated by Spatiotemporal Differences in Macrophage Subtypes. Diabetes (2008) 57(12):3239–46. 10.2337/db08-0872 PMC2584129 18829989

[21] Wu D Molofsky AB Liang HE Ricardo-Gonzalez RR Jouihan HA Bando JK. Eosinophils Sustain Adipose Alternatively Activated Macrophages Associated With Glucose Homeostasis. Science (2011) 332(6026):243–7. 10.1126/science.1201475 PMC3144160 21436399

[22] Elgazar-Carmon V Rudich A Hadad N Levy R. Neutrophils Transiently Infiltrate Intra-Abdominal Fat Early in the Course of High-Fat Feeding. J Lipid Res (2008) 49(9):1894–903. 10.1194/jlr.M800132-JLR200 18503031

[23] Nishimura S Manabe I Nagasaki M Eto K Yamashita H Ohsugi M. CD8+ Effector T Cells Contribute to Macrophage Recruitment and Adipose Tissue Inflammation in Obesity. Nat Med (2009) 15(8):914–20. 10.1038/nm.1964 19633658

[24] Kloting N Bluher M. Adipocyte Dysfunction, Inflammation and Metabolic Syndrome. Rev Endocr Metab Disord (2014) 15(4):277–87. 10.1007/s11154-014-9301-0 25344447

[25] Wang ZV Scherer PE. Adiponectin, the Past Two Decades. J Mol Cell Biol (2016) 8(2):93–100. 10.1093/jmcb/mjw011 26993047 PMC4816148

[26] Hotamisligil GS Arner P Atkinson RL Spiegelman BM. Differential Regulation of the p80 Tumor Necrosis Factor Receptor in Human Obesity and Insulin Resistance. Diabetes (1997) 46(3):451–5. 10.2337/diab.46.3.451 9032102

[27] Hotamisligil GS Arner P Caro JF Atkinson RL Spiegelman BM. Increased Adipose Tissue Expression of Tumor Necrosis Factor-Alpha in Human Obesity and Insulin Resistance. J Clin Invest (1995) 95(5):2409–15. 10.1172/JCI117936 PMC295872 7738205

[28] Hotamisligil GS Shargill NS Spiegelman BM. Adipose Expression of Tumor Necrosis Factor-Alpha: Direct Role in Obesity-Linked Insulin Resistance. Science (1993) 259(5091):87–91. 10.1126/science.7678183 7678183

[29] Kern PA Saghizadeh M Ong JM Bosch RJ Deem R Simsolo RB. The Expression of Tumor Necrosis Factor in Human Adipose Tissue. Regulation by Obesity, Weight Loss, and Relationship to Lipoprotein Lipase. J Clin Invest (1995) 95(5):2111–9. 10.1172/JCI117899 PMC295809 7738178

[30] Hug C Lodish HF. Visfatin: A New Adipokine. Science (2005) 307(5708):366. 10.1126/science.1106933 15604359

[31] Steppan CM Bailey ST Bhat S Brown EJ Banerjee RR Wright CM. The Hormone Resistin Links Obesity to Diabetes. Nature (2001) 409(6818):307–12. 10.1038/35053000 11201732

[32] Rodriguez A Ezquerro S Mendez-Gimenez L Becerril S Fruhbeck G. Revisiting the Adipocyte: A Model for Integration of Cytokine Signaling in the Regulation of Energy Metabolism. Am J Physiol Endocrinol Metab (2015) 309(8):E691–714. 10.1152/ajpendo.00297.2015 26330344

[33] Golden PL Maccagnan TJ Pardridge WM. Human Blood-Brain Barrier Leptin Receptor. Binding and Endocytosis in Isolated Human Brain Microvessels. J Clin Invest (1997) 99(1):14–8. 10.1172/JCI119125 PMC507761 9011568

[34] Zhang Y Proenca R Maffei M Barone M Leopold L Friedman JM. Positional Cloning of the Mouse Obese Gene and Its Human Homologue. Nature (1994) 372(6505):425–32. 10.1038/372425a0 7984236

[35] Campfield LA Smith FJ Guisez Y Devos R Burn P. Recombinant Mouse OB Protein: Evidence for a Peripheral Signal Linking Adiposity and Central Neural Networks. Science (1995) 269(5223):546–9. 10.1126/science.7624778 7624778

[36] Considine RV Sinha MK Heiman ML Kriauciunas A Stephens TW Nyce MR. Serum Immunoreactive-Leptin Concentrations in Normal-Weight and Obese Humans. N Engl J Med (1996) 334(5):292–5. 10.1056/NEJM199602013340503 8532024

[37] Crujeiras AB Carreira MC Cabia B Andrade S Amil M Casanueva FF. Leptin Resistance in Obesity: An Epigenetic Landscape. Life Sci (2015) 140:57–63. 10.1016/j.lfs.2015.05.003 25998029

[38] Takahashi N Waelput W Guisez Y. Leptin is an Endogenous Protective Protein Against the Toxicity Exerted by Tumor Necrosis Factor. J Exp Med (1999) 189(1):207–12. 10.1084/jem.189.1.207-a PMC1887698 9874578

[39] Faggioni R Fantuzzi G Gabay C Moser A Dinarello CA Feingold KR. Leptin Deficiency Enhances Sensitivity to Endotoxin-Induced Lethality. Am J Physiol (1999) 276(1):R136–42. 10.1152/ajpregu.1999.276.1.R136 9887187

[40] Mancuso P Gottschalk A Phare SM Peters-Golden M Lukacs NW Huffnagle GB. Leptin-Deficient Mice Exhibit Impaired Host Defense in Gram-Negative Pneumonia. J Immunol (2002) 168(8):4018–24. 10.4049/jimmunol.168.8.4018 11937559

[41] Gainsford T Willson TA Metcalf D Handman E McFarlane C Ng A. Leptin can Induce Proliferation, Differentiation, and Functional Activation of Hemopoietic Cells. Proc Natl Acad Sci (1996) 93(25):14564–8. 10.1073/pnas.93.25.14564 PMC26173 8962092

[42] Caldefie-Chezet F Poulin A Tridon A Sion B Vasson MP. Leptin: A Potential Regulator of Polymorphonuclear Neutrophil Bactericidal Action? J Leukoc Biol (2001) 69(3):414–8. 10.1189/jlb.69.3.414 11261788

[43] Bruno A Conus S Schmid I Simon HU. Apoptotic Pathways Are Inhibited by Leptin Receptor Activation in Neutrophils. J Immunol (2005) 174(12):8090–6. 10.4049/jimmunol.174.12.8090 15944317

[44] Ottonello L Gnerre P Bertolotto M Mancini M Dapino P Russo R. Leptin as a Uremic Toxin Interferes With Neutrophil Chemotaxis. J Am Soc Nephrol (2004) 15(9):2366–72. 10.1097/01.ASN.0000139321.98029.40 15339985

[45] Zhao S Zhu Y Schultz RD Li N He Z Zhang Z. Partial Leptin Reduction as an Insulin Sensitization and Weight Loss Strategy. Cell Metab (2019) 30(4):706–19.e6. 10.1016/j.cmet.2019.08.005 31495688 PMC6774814

[46] Kern PA Di Gregorio GB Lu T Rassouli N Ranganathan G. Adiponectin Expression From Human Adipose Tissue: Relation to Obesity, Insulin Resistance, and Tumor Necrosis Factor-Alpha Expression. Diabetes (2003) 52(7):1779–85. 10.2337/diabetes.52.7.1779 12829646

[47] Kim JY van de Wall E Laplante M Azzara A Trujillo ME Hofmann SM. Obesity-Associated Improvements in Metabolic Profile Through Expansion of Adipose Tissue. J Clin Invest (2007) 117(9):2621–37. 10.1172/JCI31021 PMC1950456 17717599

[48] Ohashi K Parker JL Ouchi N Higuchi A Vita JA Gokce N. Adiponectin Promotes Macrophage Polarization Toward an Anti-Inflammatory Phenotype. J Biol Chem (2010) 285(9):6153–60. 10.1074/jbc.M109.088708 PMC2825410 20028977

[49] Trellakis S Rydleuskaya A Fischer C Canbay A Tagay S Scherag A. Low Adiponectin, High Levels of Apoptosis and Increased Peripheral Blood Neutrophil Activity in Healthy Obese Subjects. Obes Facts (2012) 5(3):305–18. 10.1159/000339452 22722748

[50] Chedid P Hurtado-Nedelec M Marion-Gaber B Bournier O Hayem G Gougerot-Pocidalo MA. Adiponectin and Its Globular Fragment Differentially Modulate the Oxidative Burst of Primary Human Phagocytes. Am J Pathol (2012) 180(2):682–92. 10.1016/j.ajpath.2011.10.013 22119038

[51] Chung HS Choi KM. Adipokines and Myokines: A Pivotal Role in Metabolic and Cardiovascular Disorders. Curr Med Chem (2018) 25(20):2401–15. 10.2174/0929867325666171205144627 29210643

[52] Funcke JB Scherer PE. Beyond Adiponectin and Leptin: Adipose Tissue-Derived Mediators of Inter-Organ Communication. J Lipid Res (2019) 60(10):1648–84. 10.1194/jlr.R094060 PMC6795086 31209153

[53] Yore MM Syed I Moraes-Vieira PM Zhang T Herman MA Homan EA. Discovery of a Class of Endogenous Mammalian Lipids With Anti-Diabetic and Anti-Inflammatory Effects. Cell (2014) 159(2):318–32. 10.1016/j.cell.2014.09.035 PMC4260972 25303528

[54] Flaherty SE Grijalva A Xu X Ables E Nomani A Ferrante AW. A Lipase-Independent Pathway of Lipid Release and Immune Modulation by Adipocytes. Science (2019) 363(6430):989–93. 10.1126/science.aaw2586 PMC6579605 30819964

[55] Kita S Maeda N Shimomura I. Interorgan Communication by Exosomes, Adipose Tissue, and Adiponectin in Metabolic Syndrome. J Clin Invest (2019) 129(10):4041–9. 10.1172/JCI129193 PMC6763291 31483293

[56] Thomou T Mori MA Dreyfuss JM Konishi M Sakaguchi M Wolfrum C. Adipose-Derived Circulating miRNAs Regulate Gene Expression in Other Tissues. Nature (2017) 542(7642):450–5. 10.1038/nature21365 PMC5330251 28199304

[57] van der Linden M Meyaard L. Fine-Tuning Neutrophil Activation: Strategies and Consequences. Immunol Lett (2016) 178:3–9. 10.1016/j.imlet.2016.05.015 27262927

[58] Doeing DC Borowicz JL Crockett ET. Gender Dimorphism in Differential Peripheral Blood Leukocyte Counts in Mice Using Cardiac, Tail, Foot, and Saphenous Vein Puncture Methods. BMC Clin Pathol (2003) 3(1):3. 10.1186/1472-6890-3-3 12971830 PMC201031

[59] Mestas J Hughes CC. Of Mice and Not Men: Differences Between Mouse and Human Immunology. J Immunol (2004) 172(5):2731–8. 10.4049/jimmunol.172.5.2731 14978070

[60] Talukdar S Oh DY Bandyopadhyay G Li D Xu J McNelis J. Neutrophils Mediate Insulin Resistance in Mice Fed a High-Fat Diet Through Secreted Elastase. Nat Med (2012) 18(9):1407–12. 10.1038/nm.2885 PMC3491143 22863787

[61] Wang Q Xie Z Zhang W Zhou J Wu Y Zhang M. Myeloperoxidase Deletion Prevents High-Fat Diet-Induced Obesity and Insulin Resistance. Diabetes (2014) 63(12):4172–85. 10.2337/db14-0026 PMC4238009 25024373

[62] Braster Q Silvestre Roig C Hartwig H Beckers L den Toom M Doring Y. Inhibition of NET Release Fails to Reduce Adipose Tissue Inflammation in Mice. PloS One (2016) 11(10):e0163922. 10.1371/journal.pone.0163922 27701440 PMC5049774

[63] Shah TJ Leik CE Walsh SW. Neutrophil Infiltration and Systemic Vascular Inflammation in Obese Women. Reprod Sci (2010) 17(2):116–24. 10.1177/1933719109348252 PMC2832323 19820230

[64] Rouault C Pellegrinelli V Schilch R Cotillard A Poitou C Tordjman J. Roles of Chemokine Ligand-2 (CXCL2) and Neutrophils in Influencing Endothelial Cell Function and Inflammation of Human Adipose Tissue. Endocrinology (2013) 154(3):1069–79. 10.1210/en.2012-1415 23372021

[65] Bruun JM Pedersen SB Richelsen B. Regulation of Interleukin 8 Production and Gene Expression in Human Adipose Tissue In Vitro. J Clin Endocrinol Metab (2001) 86(3):1267–73. 10.1210/jc.86.3.1267 11238519

[66] Makki K Froguel P Wolowczuk I. Adipose Tissue in Obesity-Related Inflammation and Insulin Resistance: Cells, Cytokines, and Chemokines. ISRN Inflamm 2013 (2013) p:139239. 10.1155/2013/139239 PMC3881510 24455420

[67] Girbl T Lenn T Perez L Rolas L Barkaway A Thiriot A. Distinct Compartmentalization of the Chemokines CXCL1 and CXCL2 and the Atypical Receptor ACKR1 Determine Discrete Stages of Neutrophil Diapedesis. Immunity (2018) 49(6):1062–1076.e6. 10.1016/j.immuni.2018.09.018 30446388 PMC6303217

[68] Tynan GA Hearnden CH Oleszycka E Lyons CL Coutts G O’Connell J. Endogenous Oils Derived From Human Adipocytes Are Potent Adjuvants That Promote IL-1α-Dependent Inflammation. Diabetes (2014) 63(6):2037–50. 10.2337/db13-1476 24458363

[69] Freigang S Ampenberger F Weiss A Kanneganti T-D Iwakura Y Hersberger M. Fatty Acid–Induced Mitochondrial Uncoupling Elicits Inflammasome-Independent IL-1α and Sterile Vascular Inflammation in Atherosclerosis. Nat Immunol (2013) 14(10):1045–53. 10.1038/ni.2704 23995233

[70] Watanabe Y Nagai Y Honda H Okamoto N Yanagibashi T Ogasawara M. Bidirectional Crosstalk Between Neutrophils and Adipocytes Promotes Adipose Tissue Inflammation. FASEB J (2019) 33(11):11821–35. 10.1096/fj.201900477RR 31355683

[71] Weisberg SP McCann D Desai M Rosenbaum M Leibel RL Ferrante AW Jr. Obesity is Associated With Macrophage Accumulation In Adipose Tissue. J Clin Invest (2003) 112(12):1796–808. 10.1172/JCI200319246 PMC296995 14679176

[72] Patsouris D Li PP Thapar D Chapman J Olefsky JM Neels JG. Ablation of CD11c-Positive Cells Normalizes Insulin Sensitivity in Obese Insulin Resistant Animals. Cell Metab (2008) 8(4):301–9. 10.1016/j.cmet.2008.08.015 PMC2630775 18840360

[73] Guo L Akahori H Harari E Smith SL Polavarapu R Karmali V. CD163+ Macrophages Promote Angiogenesis and Vascular Permeability Accompanied by Inflammation in Atherosclerosis. J Clin Invest (2018) 128(3):1106–24. 10.1172/JCI93025 PMC5824873 29457790

[74] Weisberg SP Hunter D Huber R Lemieux J Slaymaker S Vaddi K. CCR2 Modulates Inflammatory and Metabolic Effects of High-Fat Feeding. J Clin Invest (2006) 116(1):115–24. 10.1172/JCI24335 PMC1307559 16341265

[75] Kanda H Tateya S Tamori Y Kotani K Hiasa K Kitazawa R. MCP-1 Contributes to Macrophage Infiltration Into Adipose Tissue, Insulin Resistance, and Hepatic Steatosis in Obesity. J Clin Invest (2006) 116(6):1494–505. 10.1172/JCI26498 PMC1459069 16691291

[76] Odegaard JI Ricardo-Gonzalez RR Goforth MH Morel CR Subramanian V Mukundan L. Macrophage-Specific PPARgamma Controls Alternative Activation and Improves Insulin Resistance. Nature (2007) 447(7148):1116–20. 10.1038/nature05894 PMC2587297 17515919

[77] Hevener AL Olefsky JM Reichart D Nguyen MT Bandyopadyhay G Leung HY. Macrophage PPAR Gamma is Required for Normal Skeletal Muscle and Hepatic Insulin Sensitivity and Full Antidiabetic Effects of Thiazolidinediones. J Clin Invest (2007) 117(6):1658–69. 10.1172/JCI31561 PMC1868788 17525798

[78] Fjeldborg K Pedersen SB Møller HJ Christiansen T Bennetzen M Richelsen B. Human Adipose Tissue Macrophages are Enhanced But Changed to An Anti-Inflammatory Profile in Obesity. J Immunol Res (2014) 2014:309548. 10.1155/2014/309548 24741586 PMC3987875

[79] Wentworth JM Naselli G Brown WA Doyle L Phipson B Smyth GK. Pro-Inflammatory CD11c(+)CD206(+) Adipose Tissue Macrophages Are Associated With Insulin Resistance in Human Obesity. Diabetes (2010) 59(7):1648–56. 10.2337/db09-0287 PMC2889764 20357360

[80] Aron-Wisnewsky J Tordjman J Poitou C Darakhshan F Hugol D Basdevant A. Human Adipose Tissue Macrophages: M1 and M2 Cell Surface Markers in Subcutaneous and Omental Depots and After Weight Loss. J Clin Endocrinol Metab (2009) 94(11):4619–23. 10.1210/jc.2009-0925 19837929

[81] Engin AB. Adipocyte-Macrophage Cross-Talk in Obesity. Adv Exp Med Biol (2017) 960:327–43. 10.1007/978-3-319-48382-5_14 28585206

[82] Suganami T Nishida J Ogawa Y. A Paracrine Loop Between Adipocytes and Macrophages Aggravates Inflammatory Changes: Role of Free Fatty Acids and Tumor Necrosis Factor Alpha. Arterioscler Thromb Vasc Biol (2005) 25(10):2062–8. 10.1161/01.ATV.0000183883.72263.13 16123319

[83] Suganami T Tanimoto-Koyama K Nishida J Itoh M Yuan X Mizuarai S. Role of the Toll-like Receptor 4/NF-Kappab Pathway in Saturated Fatty Acid-Induced Inflammatory Changes in the Interaction Between Adipocytes and Macrophages. Arterioscler Thromb Vasc Biol (2007) 27(1):84–91. 10.1161/01.ATV.0000251608.09329.9a 17082484

[84] Spalding KL Arner E Westermark PO Bernard S Buchholz BA Bergmann O. Dynamics of Fat Cell Turnover in Humans. Nature (2008) 453(7196):783–7. 10.1038/nature06902 18454136

[85] Sárvári AK Doan-Xuan QM Bacsó Z Csomós I Balajthy Z Fésüs L. Interaction of Differentiated Human Adipocytes With Macrophages Leads to Trogocytosis and Selective IL-6 Secretion. Cell Death Dis (2015) 6(1):e1613–3. 10.1038/cddis.2014.579 PMC4669775 25611388

[86] Hribal ML Fiorentino TV Sesti G. Role of C Reactive Protein (CRP) in Leptin Resistance. Curr Pharm Design (2014) 20(4):609–15. 10.2174/13816128113199990016 PMC4155811 23688010

[87] Sikaris KA. The Clinical Biochemistry of Obesity. Clin Biochemist Rev (2004) 25(3):165–81. PMC1880830 18458706

[88] Maurizi G Della Guardia L Maurizi A Poloni A. Adipocytes Properties and Crosstalk With Immune System in Obesity-Related Inflammation. J Cell Physiol (2018) 233(1):88–97. 10.1002/jcp.25855 28181253

[89] Spits H Artis D Colonna M Diefenbach A Di Santo JP Eberl G. Innate Lymphoid Cells–A Proposal for Uniform Nomenclature. Nat Rev Immunol (2013) 13(2):145–9. 10.1038/nri3365 23348417

[90] Artis D Spits H. The Biology of Innate Lymphoid Cells. Nature (2015) 517(7534):293–301. 10.1038/nature14189 25592534

[91] Hoyler T Klose CS Souabni A Turqueti-Neves A Pfeifer D Rawlins EL. The Transcription Factor GATA-3 Controls Cell Fate and Maintenance of Type 2 Innate Lymphoid Cells. Immunity (2012) 37(4):634–48. 10.1016/j.immuni.2012.06.020 PMC3662874 23063333

[92] Molofsky AB Nussbaum JC Liang H-E Van Dyken SJ Cheng LE Mohapatra A. Innate Lymphoid Type 2 Cells Sustain Visceral Adipose Tissue Eosinophils and Alternatively Activated Macrophages. J Exp Med (2013) 210(3):535–49. 10.1084/jem.20121964 PMC3600903 23420878

[93] Brestoff JR Kim BS Saenz SA Stine RR Monticelli LA Sonnenberg GF. Group 2 Innate Lymphoid Cells Promote Beiging of White Adipose Tissue and Limit Obesity. Nature (2015) 519(7542):242–6. 10.1038/nature14115 PMC4447235 25533952

[94] Lee M-W. Odegaard JI Mukundan L Qiu Y. Molofsky AB. Nussbaum JC. Activated Type 2 Innate Lymphoid Cells Regulate Beige Fat Biogenesis. Cell (2015) 160(1):74–87. 10.1016/j.cell.2014.12.011 25543153 PMC4297518

[95] Bolus WR Hasty AH. Contributions of Innate Type 2 Inflammation to Adipose Function. J Lipid Res (2019) 60(10):1698–709. 10.1194/jlr.R085993 PMC6795076 29891508

